# Fractal dimension analysis as an easy computational approach to improve breast cancer histopathological diagnosis

**DOI:** 10.1186/s42649-021-00055-w

**Published:** 2021-04-30

**Authors:** Lucas Glaucio da Silva, Waleska Rayanne Sizinia da Silva Monteiro, Tiago Medeiros de Aguiar Moreira, Maria Aparecida Esteves Rabelo, Emílio Augusto Campos Pereira de Assis, Gustavo Torres de Souza

**Affiliations:** 1 Faculty of Medical and Health Sciences of Juiz de Fora, Alameda Salvaterra, Juiz de Fora, Minas Gerais 200 – 36033-003 Brazil; 2 Department of Biology - Genetics - Federal University of Juiz de Fora, Rua José Lourenço Kelmer, s/n, Juiz de Fora, Minas Gerais 36036-900 Brazil; 3 Animal Reproduction Laboratory - Brazilian Agricultural Research Corporation – Dairy Cattle, Laboratory of Animal Reproduction, Av. Eugênio do Nascimento, Juiz de Fora, Minas Gerais 610 - 36038-330 Brazil; 4 Center for Investigation and Diagnosis of Pathological Anatomy, Avenida Itamar Franco, Juiz de Fora, Minas Gerais 4001 – 36033-318 Brazil

**Keywords:** Histopathology, Computer-aided diagnosis, Breast cancer, And fractal dimension

## Abstract

Histopathology is a well-established standard diagnosis employed for the majority of malignancies, including breast cancer. Nevertheless, despite training and standardization, it is considered operator-dependent and errors are still a concern. Fractal dimension analysis is a computational image processing technique that allows assessing the degree of complexity in patterns. We aimed here at providing a robust and easily attainable method for introducing computer-assisted techniques to histopathology laboratories. Slides from two databases were used: A) Breast Cancer Histopathological; and B) Grand Challenge on Breast Cancer Histology. Set A contained 2480 images from 24 patients with benign alterations, and 5429 images from 58 patients with breast cancer. Set B comprised 100 images of each type: normal tissue, benign alterations, in situ carcinoma, and invasive carcinoma. All images were analyzed with the FracLac algorithm in the ImageJ computational environment to yield the box count fractal dimension (Db) results. Images on set A on 40x magnification were statistically different (*p* = 0.0003), whereas images on 400x did not present differences in their means. On set B, the mean Db values presented promissing statistical differences when comparing. Normal and/or benign images to in situ and/or invasive carcinoma (all *p* < 0.0001). Interestingly, there was no difference when comparing normal tissue to benign alterations. These data corroborate with previous work in which fractal analysis allowed differentiating malignancies. Computer-aided diagnosis algorithms may beneficiate from using Db data; specific Db cut-off values may yield ~ 99% specificity in diagnosing breast cancer. Furthermore, the fact that it allows assessing tissue complexity, this tool may be used to understand the progression of the histological alterations in cancer.

## Introduction

Breast cancer is the most incident malignancy in women across the world (Cardoso et al. 2017). Despite the great variability at the molecular level, this cancer is curable in 70% to 80% of the early-stage cases (Cardoso et al. 2017; Harbeck et al. 2019). The possibility of remission underscores the importance of correct and rapid diagnosis. Although screening and initial detection rely on imaging techniques and clinical features, the final diagnosis is majorly reached upon needle biopsy followed by histopathological analysis (Irwig et al. 2002; Harbeck et al. 2019).

Histopathology is a powerful tool in the diagnosis of breast cancer, as well as it is the standard diagnosis in the great majority of other malignancies. As for breast alterations, pathological investigation and report have been well-established; World Health Organization (WHO) provides constant updates on the classification of the pathological entities regarding breast cancers (Sinn and Kreipe 2013). Furthermore, the histopathological diagnosis and further classification of breast cancer are at the core of prognosis and treatment decisions (Sinn and Kreipe 2013). Nevertheless, despite training and standardization, it is considered operator-dependent and subject to errors (Nguyen et al. 2004; Chan and Tuszynski 2016). Recent results have shown interobserver classifications of breast cancer pathological samples to be considerably good (Rabe et al. 2019); but some discrepancies still may be found (Bueno-de-Mesquita et al. 2010). Considering the possibility of interobserver differences when diagnosing malignancies, attempts to automate or employ algorithms to aid diagnosis gain attention. Computer-aided diagnosis, thus, aims at maximizing the reliability of the histopathological assessment (Chan and Tuszynski 2016). The ability to either fully diagnose or allow excluding images with specific features may be of great interest to improve the final diagnostic made by the pathology professional.

Image processing techniques have shown success in improving cancer diagnosis through several mechanisms. Recent advances have been made into classifying histopathological images of malignancies (Mohammadzadeh et al. 2015); machine learning algorithms have shown to be promising tools (Benzheng Wei et al. 2017; Komura and Ishikawa 2018; Dimitriou et al. 2019; Iizuka et al. 2020). Apart from microscopy, other sources of images have also yielded possible means for diagnosing several types of cancers, i.e.: radiological results (Li et al. 2017; Adel et al. 2019; Hu et al. 2020). Emphasis should be given to the recent results produced by Shen et al., who successfully improved detection by analyzing mammograms through their deep learning algorithms (Shen et al. 2019).

The rationale underlying the automated or computer-aided cancer detection involves, at least: 1) image preprocessing; 2) extracting identifiable features; and 3) correlating the features to the diagnostic. In this scenario, the identification of parameters that could successfully differentiate cancer images and, thus, aid in the diagnosis are of great interest (Chan and Tuszynski 2016; Angel Arul Jothi and Mary Anita Rajam 2017). The fractal dimension (FD) has been employed in several initial works as a possible manner of differentiating malignant tissue in microscopy images (Cross and Cotton 1992; Tambasco et al. 2010; Braverman and Tambasco 2013; de Arruda et al. 2013; Waliszewski et al. 2015; Maipas et al. 2018). FD is, ultimately, a mathematical entity capable of determining the complexity of two-dimensional objects. The computation analysis of complex objects largely benefits from being understood under the lenses of fractal geometry (Nayak et al. 2019).

Carcinogenesis involves, as per definition, among other characteristics, impaired cellular proliferation control and the capacity of invading tissue (Hanahan and Weinberg 2000). These hallmarks imply that the formation of a tumor generally progresses with distortion of the tissue architecture, altering its complexity in comparison to the healthy counterpart. Such notions, in addition to the previous works in which FD was used, have guided us to further study the utility of FD in diagnosing malignancies in histopathological slides from different sources.

Although the fractal analysis involves a complex concept, we aim here to provide a user-friendly computational protocol able to assess the presence of malignancies in breast tissue slides. The underlying theory of the present work is that the tissue architecture is sufficiently altered during carcinogenesis to be detected by fractal analysis, as the complexity of the tissue is quantifiable with this technique.

## Material and methods

### Datasets used for analysis

In the present work, we aimed at establishing the performance of FD as a feature capable of distinguishing breast carcinomas from normal tissue and benign alterations. With that purpose, we employed images from two different databases: a) Breast Cancer Histopathological Database (BreakHis – available at: https://web.inf.ufpr.br/vri/databases/breast-cancer-histopathological-database-breakhis/) (Spanhol et al. 2016); and b) Grand challenge on breast cancer histology images (BACH – available at: https://iciar2018-challenge.grand-challenge.org/download/) (Aresta et al. 2019). Both sets were generated from clinical specimens stained with hematoxylin and eosin and are available on the respective websites for download and consultation. All slides received previous histopathological diagnostic as described by the authors who made them available (Spanhol et al. 2016; Aresta et al. 2019). Tables 1 and 2 summarize the diagnostics. Characteristics regarding the composition and peculiarities of each database will be discussed in the results section.

### Computational environment and box count fractal dimension measurement on ImageJ

We performed all measurements using the FracLac algorithm on ImageJ. This computational environment allows for easy measurement of the fractal characteristics of the images while not requiring expertise in programming. Furthermore, the FracLac plugin allows analyzing images as a batch. The FracLac algorithm was operated on the batch mode with the following setup: sampling sizes 3 pixels; minimum pixel size 1 pixel; the maximum percentage of the image 5%. The images were previously converted into binary by using a customized macro function on ImageJ.

So as to understand the process, results are obtained through the following path:

1. The slide images from each group and database are initially simplified into binary images;
2. The edges in the images are detected;
3. FracLac defines boxes of progressive sizes with which it gathers data and yield the box-counting fractal dimension (Db).

Considering the direct correlation between FD and the complexity of two-dimensional images, we aimed at assaying the alterations regarding complexity assumed by tissues that underwent malignant transformation. The Db was, then, our variable of interest when making all statistical comparisons.

### Statistical analysis

All calculations and graphics were produced on the GraphPad Prism 9. One-way Welch ANOVA was employed to interrogate statistical differences on all means. We compared the results within each set. A ROC curve was also calculated from both sets, so as to establish the plausibility of cut-off values (of Db) for good sensitivity and specificity. We assumed differences to be statistically significant at *p* < 0.01, however, *p*-values will be displayed for each comparison.

## Results and discussion

### Characteristics of the datasets

Both sets contain an impressive number of images derived from clinical specimens of breast cancer as well as benign alterations. The BACH set also possesses normal breast tissue slides. Thus, in our analyses, we were able to fully compare the histological status to their Db.

Considering the BreakHis set had been previously studied on the work of Chan et al. (Chan and Tuszynski 2016) with prominent results for the slides captured under 40x magnification, we have only analyzed the images on 40x and 400x magnifications. All images on the BACH set were captured under 200x magnification. The composition of each set is summarized in Tables 1 and 2 – only the images analyzed are displayed on the tables.

**Table 1:** Summarized composition of the number of images on the BreakHis set

	BreakHis (40x and 400x)							
	Benign				Malignant			
	Adenosis	Fibroadenoma	Tubular Adenoma	Phyllodes Tumor	Ductal Carcinoma	Lobular Carcinoma	Mucinous Carcinoma	Papillary Carcinoma
	114 (40x)/106 (400x)	253 (40x)/237 (400x)	109 (40x)/115 (400x)	149 (40x)/130 (400x)	864 (40x)/788 (400x)	156 (40x)/ 137 (400x)	205 (40x)/ 169 (400x)	145 (40x)/ 138 (400x)
Total	625 (40x)/588 (400x)				1370 (40x)/1232 (400x)

**Table 2:** Summarized composition of the number of images on the BACH set

	BACH (200x)			
	Normal Breast Tissue	Benign	Malignant	
			In situ	Invasive
	100	100	100	100
Total	100	100	100	100

The images on BreakHis set were derived from samples of 24 patients with benign alterations and 58 cancer patients. BreakHis images were captured with the resolution of 700 × 460 pixels, while the ones on the BACH set had the resolution of 2048 × 1536 pixels.

**Fig. 1:**
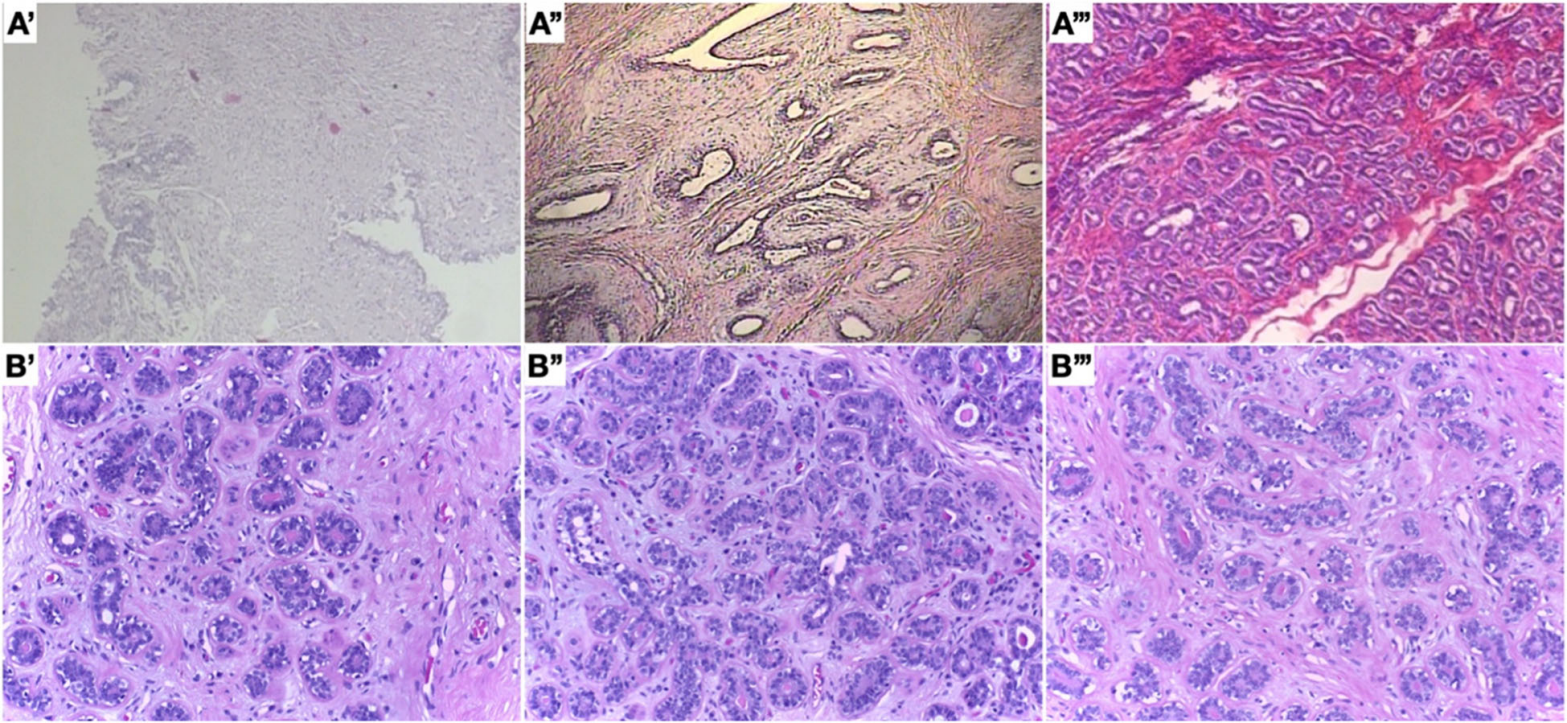
Representative images of sets BreakHis and BACH. A’-A”’) Breast tissue images from the BreakHis set captured under 40x magnification; B′-B″’) Breast tissue images from the BACH set captured under 200x magnification

Although both sets only had images captured from slides produced in a single laboratory (Spanhol et al. 2016; Aresta et al. 2019), the images on the BreakHis set were less homogenous. Upon visual inspection, the images on the BACH set displayed a more homogenous staining (Fig. 1). No direct measure for that is currently possible, however, we hypothesized it had some impact on our capacity of employing the fractal dimension to differentiate the images.

### Box count fractal dimension analysis

**Fig. 2:**
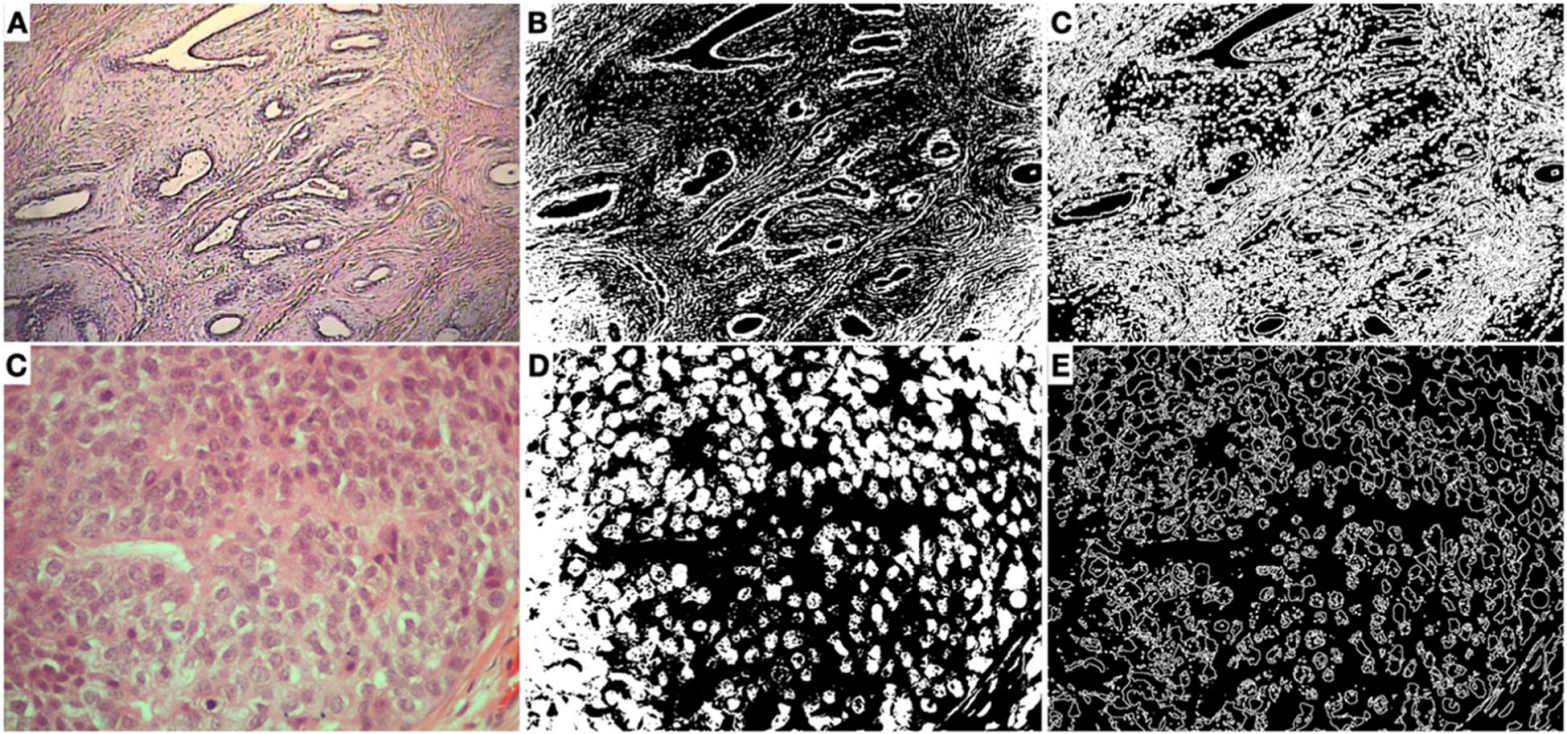
Analysis protocol with a representative image from the BACH set. **a** Benign slide from BreakHis set (40X); **b** Binarized image (40X); **c** Edge detection (40X); **d-f** Depict the protocol applied to a higher magnification image, 200X

**Fig. 3:**
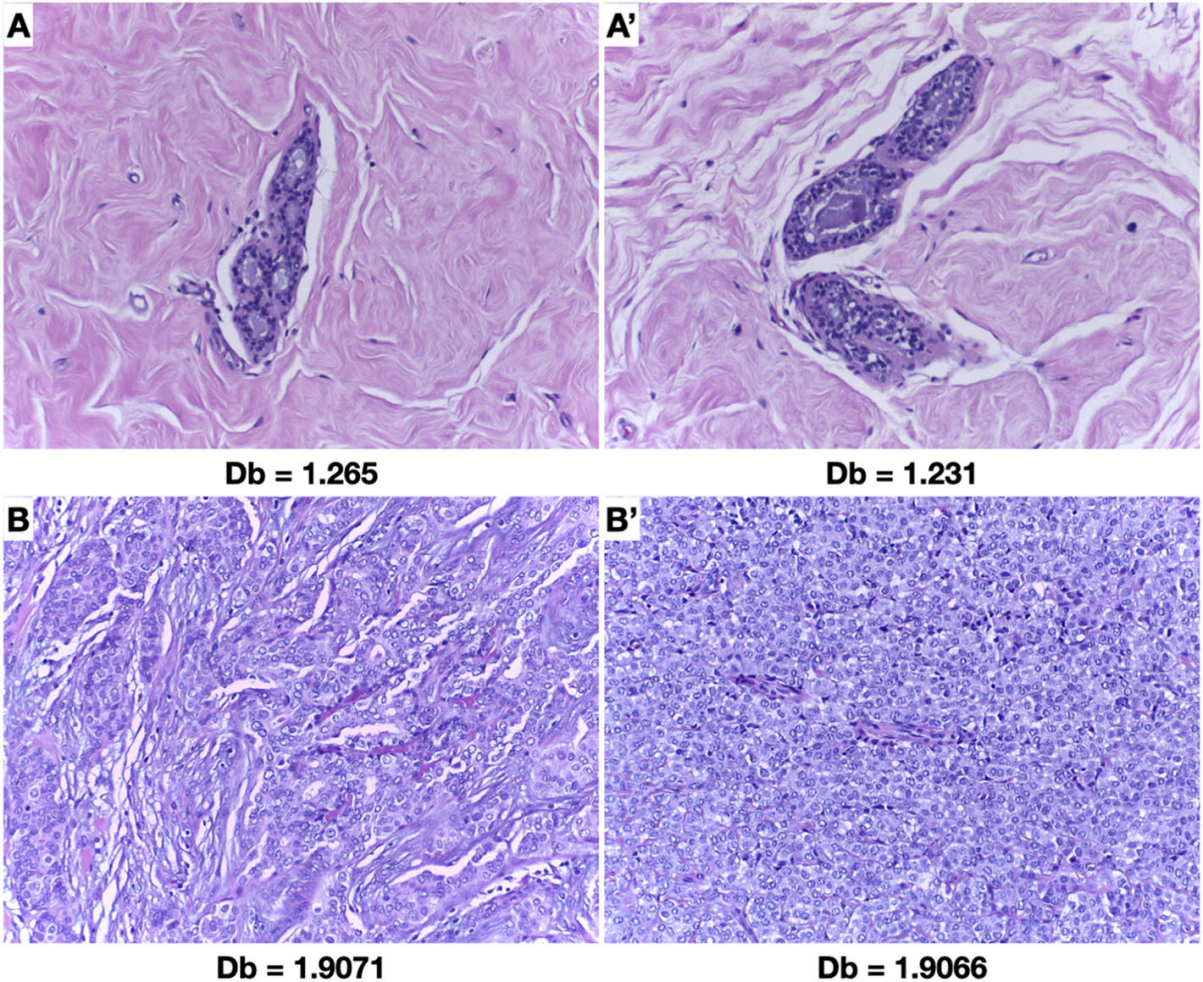
Representative comparison of Db obtained from images produced from the BACH set. A and A’: normal breast tissue; B and B′: invasive breast carcinoma

On Fig. 2, we display a schematized evolution of how the images (at 40X and 200X) are analyzed with the protocol described. Note that at a higher magnification, the edges detected are mostly from the nuclei, while the 40X image depicts the tissue architecture better. Figure 3 shows representative images associated with the box count fractal dimension (Db) associated with them.

**Fig. 4:**
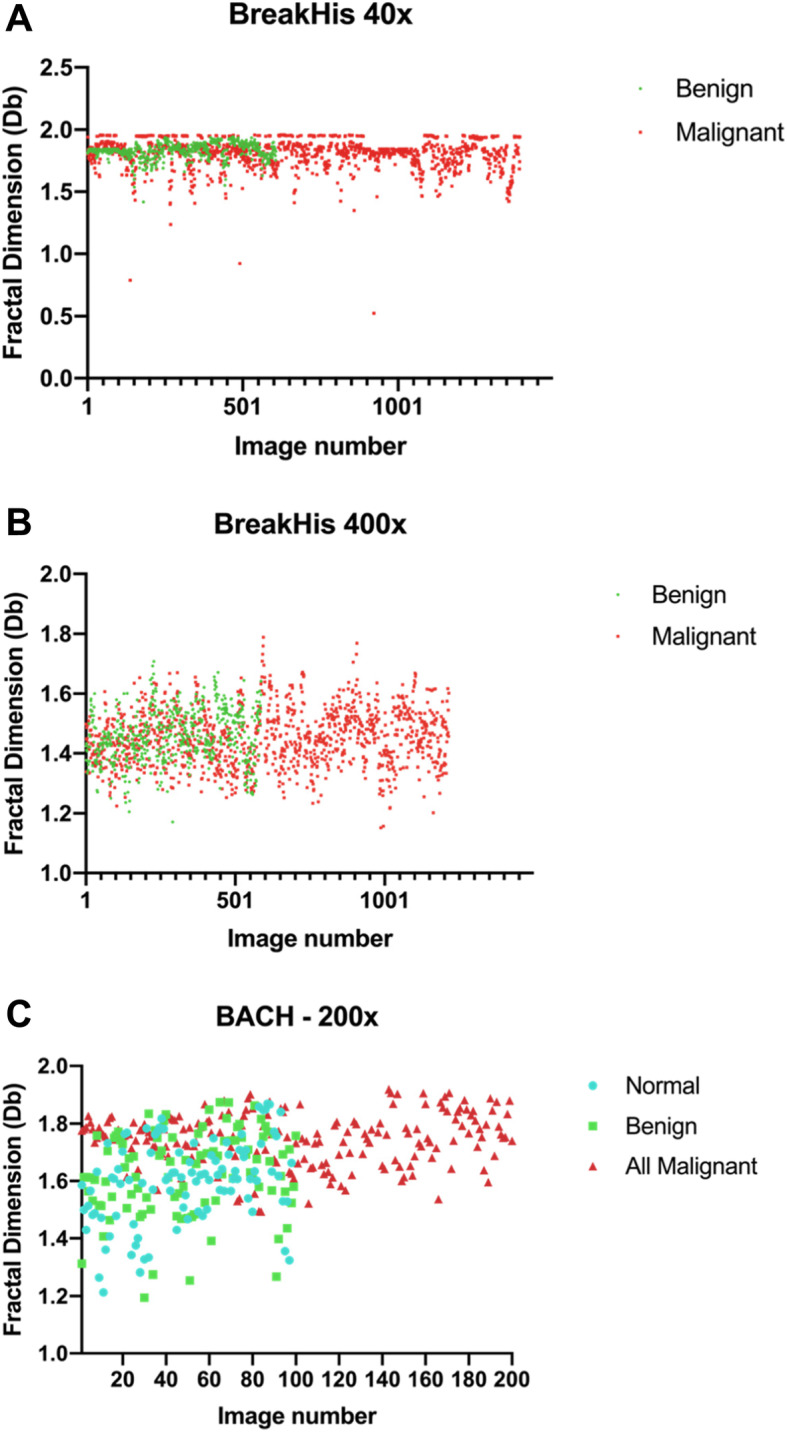
**a** Db distribution between benign and malignant images from BreakHis under 40x magnification; **b** Db distribution between benign and malignant images from BreakHis under 400x magnification; **c** Db distribution between normal tissue, benign and malignant images from set BACH

Figure 4 summarizes the distribution of Db associated with each image.

In order to start analyzing, we compared each possible group with a one-way ANOVA protocol, the main results are summarized in Table 3.

**Table 3:** Summary of the statistical analysis between each group inside each set and magnification

Set and Magnification	Group Compared	*p*-value	Statistical Significance
BreakHis – 40x	Benign vs. Malignant	0.0003	Yes
	Benign vs. Ductal CA	0.0895	No
	Benign vs. Lobular CA	0.0089	Yes
	Benign vs. Mucinous CA	< 0.0001	Yes
	Benign vs. Papillary CA	< 0.0001	Yes
	Malignant vs. Tubular Adenoma	0.2993	No
	Malignant vs. Adenosis	0.4519	No
	Malignant vs. Fibroadenoma	< 0.0001	Yes
	Malignant vs. Phyllodes Tumor	0.1533	No
BreakHis – 400x	Benign vs. Malignant	0.1452	No
	Benign vs. Ductal CA	0.0030	Yes
	Benign vs. Lobular CA	0.0047	Yes
	Benign vs. Mucinous CA	0.4533	No
	Benign vs. Papillary CA	0.4031	No
	Malignant vs. Tubular Adenoma	0.0187	No
	Malignant vs. Adenosis	0.9419	No
	Malignant vs. Fibroadenoma	0.0001	Yes
	Malignant vs. Phyllodes Tumor	0.5604	No
BACH – 200x	Normal vs. All Malignant	< 0.0001	Yes
	Normal vs. in situ CA	< 0.0001	Yes
	Normal vs. Invasive CA	< 0.0001	Yes
	Normal vs. Benign	0.0852	No
	Benign vs. in situ *CA*	< 0.0001	Yes
	Benign vs. Invasive CA	< 0.0001	Yes
	All malignant vs. Normal + Benign	< 0.0001	Yes

Statistical analysis revealed that with the BreakHis dataset, slides captured under 40x presented different mean Db when comparing benign and malignant results, whereas the 400x images did not allow this differentiation. This initial result corroborates with the study performed by Chan et al. (Chan and Tuszynski 2016). On our data, the BACH set was analyzed and presented noted statistical differences when comparing both normal and benign tissue images to malignant ones. Our primary consideration regarding the BreakHis data is that when analyzing a greater proportion of the tissue (40x), fractal analysis was able to better infer malignancy. Thus, the 400x magnification images did not yield significant differences in mean Db. Although these results are in accordance with the literature, we had considerably more promising results even when analyzing the BACH set images under 200x magnification. From that, we may hypothesize that the greater resolution and the considerable staining homogeneity (visually) among these images might be the reason for such better results. Refer to Table 3 for all *p*-values.

**Fig. 5:**
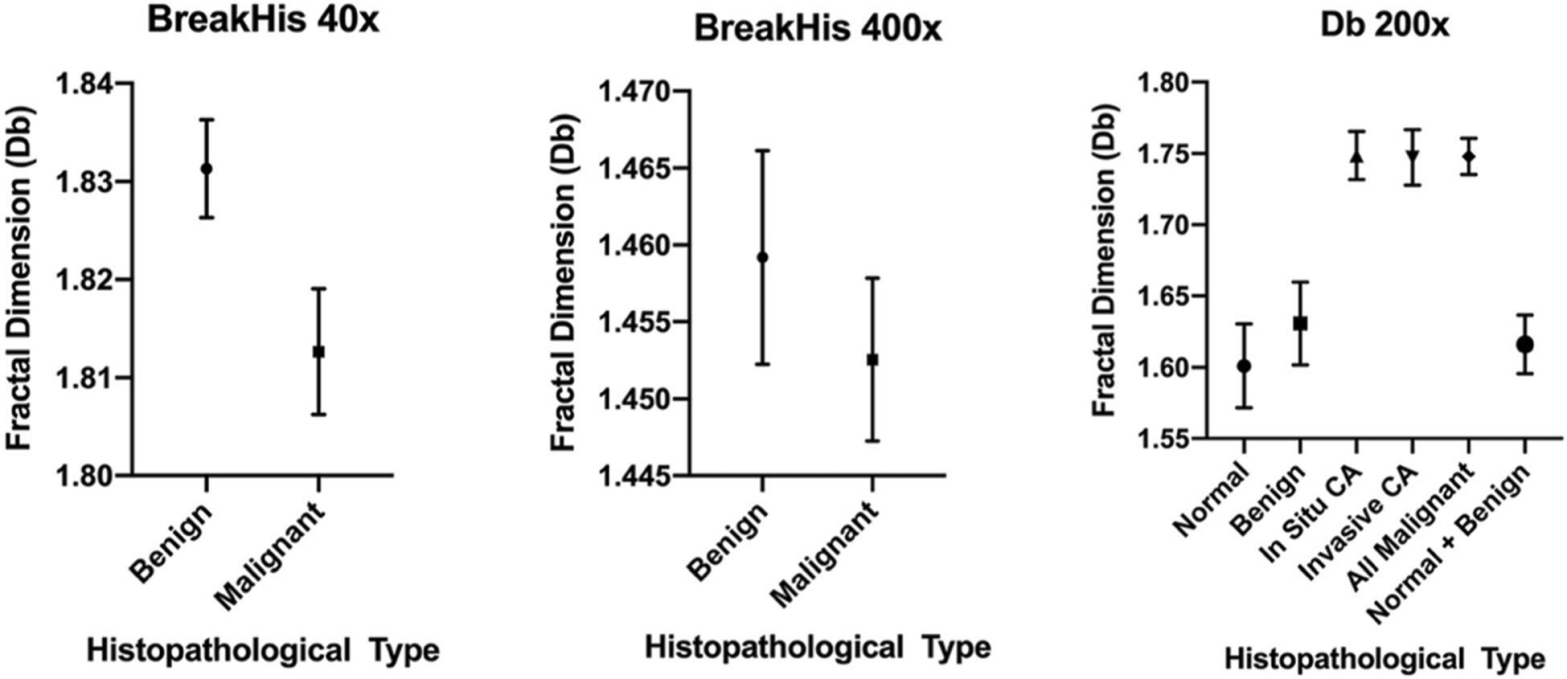
**a** One-Way ANOVA analysis results reported as mean with 95% confidence interval of the benign and malignant images from BreakHis under 40x magnification; **b** One-Way ANOVA analysis results reported as mean with 95% confidence interval of the benign and malignant images from BreakHis under 400x magnification; **c** One-Way ANOVA analysis results reported as mean with 95% confidence interval of all comparisons performed on set BACH

While malignant and benign images captured under 400x magnification in the BreakHis were not statistically different, we still noted mean Db differences between benign images and those from ductal and lobular carcinomas (*p* = 0.0030 and *p* = 0.0047, respectively). These are particularly interesting results. We may conclude that the fact that ductal and locular carcinoma implies in deeper tissue architectural alterations yielded this outcome even under 400x magnification. Figure 5 displays a graphical representation of the mean differences.

When considering only the BACH data, we had differences in the comparison between normal, benign, and both types of malignant breast alterations, in all possible combinations. Interestingly, mean Db could not differentiate normal and benign-altered tissue, which further corroborates with the notion that the fractal analysis allows for a computational-aided investigation of the tissue architecture (Klonowski et al. 2010; Chan and Tuszynski 2016). Slides assigned normal and benign results do not display important alterations on the organization of the tissue. Of note, the mean Db values for normal and benign slides on BACH had lower values than the in situ and invasive counterparts. That logic was inverted in relation to the BreakHis (40x), probably due to the higher magnification, resolution, and consistency.

**Fig. 6:**
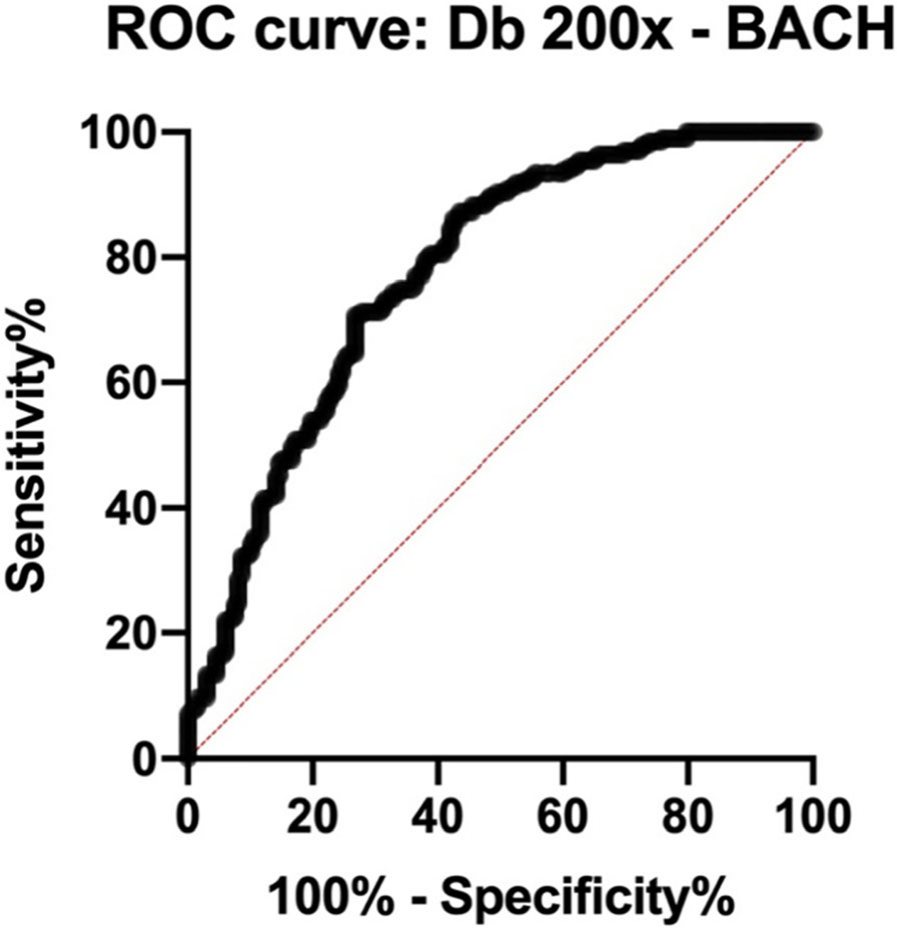
ROC curve produced from the comparison between images assigned as normal and benign and in situ and invasive carcinoma

So as to assign diagnostic power to the Db results, we performed a receiver operator characteristic (ROC) curve analysis of the data derived from the BACH set (Fig. 6).

The area under the curve was of 0.7742, with *p* < 0.0001(C.I. 99%: 0,7140 to 0,8343).

Taking only the BACH data into consideration, we may detect Db cut-off values suitable for both considerable specificity and sensitivity. From the ROC curve analysis, the Db value of 1.530 yields 98% sensitivity. Sensitivity remains above 90% until Db = 1.619.

## Conclusion

Data produced from box count fractal dimension analysis produced mean values of Db that were statistically significant when comparing malignant tissue and benign (as well as normal) of both the BreakHis set (on 40x magnification) and the BACH set.

The notion that fractal analysis succeeds in detecting the degree of complexity on images allows proper interpretation of some of our results: e.g.: 1) there was no statistical difference between normal tissue and benign breast alteration samples; and 2) on the 400x magnification slides from the BreakHis set, we still had some statistically significant difference between benign alterations, and lobular or ductal carcinomas. Nevertheless, we could not achieve classifying tumors with our technique. The capacity of differentiating samples regarding their complexity appears as an important.

The capacity of differentiating images regarding the complexity of the tissue captured figures is an important parameter for histopathological analysis. The uncontrolled proliferation and cell-to-cell signalization result in progressive architectural changes with the malignant transformation (Tambasco et al. 2010; Hanahan and Weinberg 2011). Taking all together, these results corroborate with the possibility of employing the fractal analysis as one parameter for computer-aided histopathological analysis. The fact that one of the sets yielded results calls our attention to the possibility of producing algorithms to be employed inside each laboratory, which may be fed and progressively enhanced with the addition of images. Furthermore, the fractal analysis seems to be an important parameter to be observed in other cancers, both for diagnosis and for better understanding the progression of the histopathological features.

We have performed all analyses under considerably direct and user-friendly computational conditions. Our aim here was to show this technique as a viable and useful tool for the future clinical practice of histopathology. Further studies will still be performed in order to fully standardize this technique to achieve its potential for clinical settings.

## Data Availability

All data employed in the computational evaluations are readily available online for consultation and confirmation.
